# Efficacy and safety of lurbinectedin and doxorubicin in relapsed small cell lung cancer. Results from an expansion cohort of a phase I study

**DOI:** 10.1007/s10637-020-01025-x

**Published:** 2021-03-11

**Authors:** María Eugenia Olmedo, Martin Forster, Victor Moreno, María Pilar López-Criado, Irene Braña, Michael Flynn, Bernard Doger, María de Miguel, José Antonio López-Vilariño, Rafael Núñez, Carmen Kahatt, Martin Cullell-Young, Ali Zeaiter, Emiliano Calvo

**Affiliations:** 1 Hospital Ramon y Cajal, Madrid, Spain; 2 University College of London Hospital and UCL Cancer Institute, London, UK; 3 START Madrid – FJD (Hospital Fundación Jiménez Díaz), Madrid, Spain; 4 M.D. Anderson Cancer Center, Madrid, Spain; 5 Hospital Universitari Vall d’Hebron, Barcelona, Spain; 6 START Madrid - HM CIOCC, Hospital Madrid Norte Sanchinarro, Madrid, Spain; 7 Pharma Mar, S.A., Colmenar Viejo, Madrid, Spain

**Keywords:** Lurbinectedin, PM01183, Small cell lung cancer, Phase I study

## Abstract

*Background* A phase I study found remarkable activity and manageable toxicity for doxorubicin (bolus) plus lurbinectedin (1-h intravenous [i.v.] infusion) on Day 1 every three weeks (q3wk) as second-line therapy in relapsed small cell lung cancer (SCLC). An expansion cohort further evaluated this combination. *Patients and methods* Twenty-eight patients with relapsed SCLC after no more than one line of cytotoxic-containing chemotherapy were treated: 18 (64%) with sensitive disease (chemotherapy-free interval [CTFI] ≥90 days) and ten (36%) with resistant disease (CTFI <90 days; including six with refractory disease [CTFI ≤30 days]). *Results* Ten patients showed confirmed response (overall response rate [ORR] = 36%); median progression-free survival (PFS) = 3.3 months; median overall survival (OS) = 7.9 months. ORR was 50% in sensitive disease (median PFS = 5.7 months; median OS = 11.5 months) and 10% in resistant disease (median PFS = 1.3 months; median OS = 4.6 months). The main toxicity was transient and reversible myelosuppression. Treatment-related non-hematological events (fatigue, nausea, decreased appetite, vomiting, alopecia) were mostly mild or moderate. *Conclusion* Doxorubicin 40 mg/m^2^ and lurbinectedin 2.0 mg/m^2^ on Day 1 q3wk has shown noteworthy activity in relapsed SCLC and a manageable safety profile. The combination is being evaluated as second-line therapy for SCLC in an ongoing, randomized phase III trial. *Clinical trial registration* www.ClinicalTrials.gov code: NCT01970540. Date of registration: 22 October, 2013.

## Introduction

Lurbinectedin is a selective inhibitor of oncogenic transcription that binds preferentially to guanines located in the GC-rich regulatory areas of DNA gene promoters [1, 2]. By preventing binding of transcription factors to their recognition sequences, the drug inhibits oncogenic transcription and leads to tumor cell apoptosis [3]. Lurbinectedin also affects the tumor microenvironment landscape by inhibiting activated transcription in tumor-associated macrophages [4].

Studies in mice found antitumor activity for lurbinectedin against different models of xenografted human-derived tumors. In the first-in-human (FiH) study, single-agent lurbinectedin showed antitumor activity in patients with advanced solid tumors (pancreatic cancer, soft tissue sarcoma, melanoma) [5]. The combination of doxorubicin and lurbinectedin resulted in significantly stronger antitumor effect compared to either drug alone in mice bearing xenografted small cell lung cancer (SCLC) tumors, with additive and sometimes synergistic effects [6]. This improved activity led to the conduct of a phase I trial to evaluate this combination of doxorubicin and lurbinectedin in selected advanced solid tumors, including relapsed SCLC. The recommended dose (RD) for the combination was defined at doxorubicin 50 mg/m^2^ and lurbinectedin 4.0 mg flat dose (FD) on Day 1 every three weeks (q3wk) [7]. Promising antitumor activity was found in second-line SCLC and endometrial cancer during dose escalation. However, 88% of patients treated at this RD had grade 3/4 neutropenia regardless of relationship. As a result, the combination was further evaluated in an expansion cohort at a lower doxorubicin dose to reduce the incidence of potentially severe myelosuppression. In addition, the lurbinectedin dose was transformed to a body surface area (BSA)-based dose following the finding, in a logistic regression analysis of pooled data from phase II trials with single-agent lurbinectedin, that patients with the lowest BSA values could have a greater possibility of developing grade 3/4 thrombocytopenia. This expansion cohort only enrolled patients with advanced SCLC to be treated as second-line therapy and patients with endometrial cancer. Due to the relevance of the antitumor activity observed in this cohort, the results shown here are focused on advanced SCLC patients.

## Patients and methods

### Eligibility criteria

Eligible patients were aged ≥18 years with confirmed SCLC treated with no more than one prior line of cytotoxic-containing chemotherapy regimen for advanced disease (other than anthracyclines); with documented disease progression during or immediately after last therapy; who had recovered from previous toxicities; ≥3 weeks since last anticancer therapy; Eastern Cooperative Oncology Group performance status ≤2; normal left ventricular ejection fraction (LVEF); and adequate bone marrow, hepatic and renal function, including albumin ≥3.0 g/dL.

Patients were excluded if they had symptomatic progressive or corticosteroid-requiring brain metastases/leptomeningeal involvement; were pregnant or lactating women, or were not using effective contraception; had prior bone marrow/stem cell transplantation, relevant cardiac disease, alcohol consumption or cirrhosis, active uncontrolled infection, or any disease interfering with study outcome.

### Study treatment

Patients were treated with doxorubicin 40 mg/m^2^ as bolus followed by lurbinectedin 2.0 mg/m^2^ intravenously (i.v.) over one hour on Day 1 q3wk. This dose was based on the RD defined during dose escalation (doxorubicin 50 mg/m^2^ and lurbinectedin 4.0 mg flat dose [FD]) [7], with a reduced doxorubicin dose and the lurbinectedin dose transformed to a BSA-based dose. Both drug doses were capped at a BSA of 2.0 m^2^. Patients who received ten cycles of the combination or had to discontinue doxorubicin due to a cardiac adverse event were switched to lurbinectedin alone at its single-agent RD defined during the First-in-Human study (4.0 mg/m^2^ on Day 1 q3wk) to prevent doxorubicin-induced cardiomyopathy [5, 8].

Commercially available doxorubicin was provided. Lurbinectedin was supplied as a lyophilized powder concentrate, reconstituted in sterile water for injection, and diluted with glucose 5% or sodium chloride 0.9% solution. All patients received standard antiemetic prophylaxis before each infusion. Treatment was given until disease progression, unacceptable toxicity, intercurrent illness precluding study continuation, patient refusal and/or non-compliance with study requirements, treatment delay >15 days (except if clear clinical benefit), and requirement of >2 dose reductions.

### Dose-limiting toxicities

Protocol guidelines concerning dose feasibility and definition of dose-limiting toxicities (DLTs) that were used during dose escalation in this study also applied to this cohort [7]. The regimen of doxorubicin 40 mg/m^2^ and lurbinectedin 2.0 mg/m^2^ on Day 1 q3wk would be considered feasible if less than one third of evaluable patients had DLTs during Cycle 1.

### Study assessments

Hematology and biochemistry tests were conducted at baseline, weekly during Cycle 1, and before each lurbinectedin infusion and on Day 10 during subsequent cycles. Electrocardiograms and LVEF assessments were done at baseline, and were repeated at doxorubicin discontinuation or if clinically indicated.

Antitumor activity was evaluated every two cycles according to the Response Evaluation Criteria In Solid Tumors (RECIST) v.1.1 [9]. Overall response rate (ORR) was the percentage of patients with complete (CR) or partial response (PR), and disease control rate (DCR) was the percentage of patients with response or stable disease (SD). Time-to-event parameters were duration of response (DoR), progression-free survival (PFS) and overall survival (OS).

Adverse events (AEs) and laboratory abnormalities were graded using the National Cancer Institute Common Terminology Criteria for Adverse Events (NCI-CTCAE) v.4 [10], and coded with the Medical Dictionary for Regulatory Activities (MedDRA) v.14.1.

### Statistical analysis

Continuous variables were presented with summary statistics and categorical variables in frequency tables. Time-to-event variables were calculated using Kaplan-Meier approach. Binomial exact distribution was used to calculate 95% confidence intervals (95%CIs) for categorical variables.

## Results

### Dose feasibility

Forty-seven patients with SCLC or endometrial cancer in this cohort were treated with doxorubicin 40 mg/m^2^ and lurbinectedin 2.0 mg/m^2^ on Day 1 q3wk. DLTs occurred in four of 46 (9%) evaluable patients (four of 28 [14.3%] with SCLC), thereby confirming feasibility. DLTs comprised grade 3/4 febrile neutropenia (*n* = 2), grade 4 thrombocytopenia, and grade 3 decreased appetite (*n* = 1 each).

### Characteristics of SCLC patients

Twenty-eight of the 47 patients treated in this cohort had relapsed SCLC. Most of these patients were male (*n* = 21, 75%) and had an ECOG PS of 1 (*n* = 19, 68%) (Table 1). Median age was 64 years (range, 49–77 years). At baseline, 21 (75%) had bulky disease (target lesion >50 mm). Median number of sites of disease per patient was 3 (range, 1–6 sites). Most common sites of disease were lymph nodes (*n* = 19, 68%), lung (*n* = 17, 61%), liver (*n* = 14, 50%) and bone (*n* = 9, 32%). One patient (4%) had brain metastases. Eighteen patients (64%) had sensitive disease (chemotherapy-free interval [CTFI] ≥90 days after first-line therapy) and ten patients (36%) had resistant disease (CTFI <90 days, including six patients with refractory disease [CTFI ≤30 days]).

**Table 1:** Baseline characteristics of patients with relapsed SCLC

	Doxorubicin 40 mg/m^2^ + lurbinectedin 2.0 mg/m^2^ (*n* = 28)	
	n	%
Gender		
Male	21	75
Female	7	25
Median age (range) (years)	64.0 (49–77)	
ECOG performance status		
0	9	32
1	19	68
Median BSA (range) (m^2^)	1.9 (1.5–2.3)	
Smoker		
Current	13	46
Former	14	50
Never	1	4
Median number of sites of disease involvement (range)	3.0 (1–6)	
Metastasis at baseline		
Lymph nodes	19	68
Lung	17	61
Liver	14	50
Bone	9	32
Pleura	6	21
Adrenal	5	18
CNS	1	4
Bulky disease (any target lesion > 50 mm)	21	75
Prior therapy		
Systemic therapy	28	100
Chemotherapy	28	100
Biological therapy	2	7
Radiotherapy	22	79
PCI	11	39
Prior anticancer agents		
Platinum compounds	28	100
Podophyllotoxin derivatives	28	100
CTFI		
≥90 days	18	64
<90 days	10	36
TTP from diagnosis to first infusion (months)	8.4 (3.9–19.8)	
TTP to last prior therapy (months)	6.8 (1.0–18.9)	

*BSA*, body surface area; *CNS*, central nervous system; *CTFI*, chemotherapy-free interval; *ECOG*, Eastern Cooperative Oncology Group; *PCI*, prophylactic cranial irradiation; *SCLC*, small cell lung cancer; *TTP*, time to progression

All patients received prior systemic anticancer therapy with platinum compounds and etoposide. Twenty-two patients (79%) received prior radiotherapy, including 11 patients (39%) who were given prophylactic cranial irradiation (PCI).

### Treatment exposure

Patients received 140 cycles of the combination (median: 4 cycles [range, 1–10 cycles] per patient). Median doxorubicin cumulative dose per patient was 159.9 mg/m^2^ (range, 40.0–406.4 mg/m^2^), and median relative dose intensity was 92.4% (range, 69.7–105.5%). For lurbinectedin, median cumulative dose per patient was 7.2 mg/m^2^ (range, 2.0–20.1 mg/m^2^) and median relative dose intensity was 92.4% (range, 62.3–105.5%). Six patients (21%) received 13 cycles of single-agent lurbinectedin after doxorubicin discontinuation (median: 2 cycles [range, 1–4 cycles] per patient), for a median cumulative dose of 7.6 mg/m^2^ (range, 2.0–12.8 mg/m^2^) and a median relative dose intensity of 100.2% (range, 80.2–101.2%). Most patients (*n* = 23, 82%) discontinued treatment due to radiologically confirmed disease progression; no patients discontinued as a result of treatment-related adverse events.

### Efficacy

All treated patients were evaluable for efficacy. Ten patients showed confirmed response (ORR = 36% [95%CI, 18.6–55.9%]; one CR [4%] and nine PR [32%]) and ten patients (36%) had SD, for a DCR of 72% (95%CI, 51.3–86.8%). Nine confirmed responses (one CR and eight PR) and six SD occurred among patients with sensitive disease (ORR = 50% [95%CI, 26.0–73.9%]; DCR = 83% [95%CI, 58.6–96.4%]). Only one confirmed PR and four SD were found among patients with resistant disease (ORR = 10% [95%CI, 0.25–44.5%]; DCR = 50% [95%CI, 18.7–81.3%]) (Table 2).

**Table 2:** Best tumor response according to Response Evaluation Criteria In Solid Tumors (RECIST) in patients with relapsed SCLC treated with doxorubicin 40 mg/m^2^ and lurbinectedin 2.0 mg/m^2^ on Day 1 q3wk

	Doxorubicin 40 mg/m^2^ + lurbinectedin 2.0 mg/m^2^							
	All patients with relapsed SCLC						Patients with relapsed, non-refractory SCLC ^a^ (*n* = 22)	
	CTFI ≥ 90 days (*n* = 18)		CTFI < 90 days (*n* = 10)		Total (*n* = 28)			
	n	%	n	%	n	%	n	%
CR	1	6	.	.	1	4	1	5
PR	8	44	1	10	9	32	9	41
SD	6	33	4	40	10	36	7	32
≥4 months	3	17	1	10	4	14	3	14
<4 months	3	17	3	30	6	21	4	18
PD	3	17	5	50	8	29	5	19
ORR (95%CI)	50% (26.0–73.9%)		10% (0.25–44.5%)		36% (18.6–55.9%)		46% (24.4–67.8%)	
DCR (95%CI)	83% (58.6–96.4%)		50% (18.7–81.3%)		72% (51.3–86.8%)		77% (54.6–92.2%)	
Median DoR (months) (95%CI)	5.5 (1.0–9.5)		1.8 (−)		5.2 (1.0–6.9)		5.2 (1.0–6.9)	
Median PFS (months) (95%CI)	5.7 (2.6–7.9)		1.3 (0.8–3.4)		3.3 (1.4–6.2)		5.1 (1.9–6.7)	
Median OS (months) (95%CI)	11.5 (6.0–16.6)		4.6 (0.8–6.7)		7.9 (4.2–11.5)		10.2 (6.0–11.7)	

^a^Excludes patients with CTFI ≤30 days after first-line therapy

*CI*, confidence interval; *CR*, complete response; *CTFI*, chemotherapy-free interval; *DCR*, disease control rate; *DoR*, duration of response; *ORR*, overall response rate; *OS*, overall survival; *PD*, progressive disease; *PFS*, progression-free survival; *PR*, partial response; q3wk, every three weeks; *SCLC*, small cell lung cancer; *SD*, stable disease

**Fig. 1:**
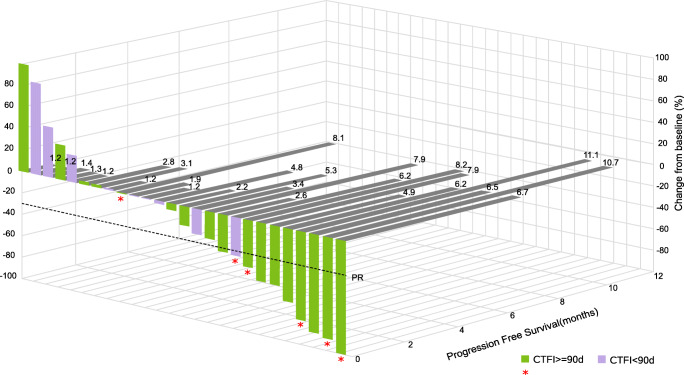
Waterfall plot showing maximum variation of target lesions and progression-free survival in patients with at least one radiological tumor assessment (*n* = 26). Ten patients had target lesion decrease >30%: one with CR and nine with PR. Red stars = treatment switch to lurbinectedin alone. CR, complete response; CTFI, chemotherapy-free interval; d, days; PR, partial response

Tumor shrinkage was observed in 18 of 26 (69%) patients with at least one radiological tumor assessment (Fig. 1): 13 of 18 (72%) with sensitive disease and five of eight (63%) with resistant disease. Antitumor activity achieved with the combination was maintained in four of six patients treated with single-agent lurbinectedin after doxorubicin discontinuation.

Median DoR was 5.2 months (95%CI, 1.0–6.9 months) in all patients (sensitive disease: 5.5 months [95%CI, 1.0–9.5 months]; the single response found in a patient with resistant disease lasted 1.8 months). In all patients, median PFS was 3.3 months (95%CI, 1.4–6.2 months) (sensitive disease: 5.7 months [95%CI, 2.6–7.9 months]; resistant disease: 1.3 months [95%CI, 0.8–3.4 months]) and median OS was 7.9 months (95%CI, 4.2–11.5 months) (sensitive disease: 11.5 months [95%CI, 6.0–16.6 months]; resistant disease: 4.6 months [range, 0.8–6.7 months]). In the 10 responders, median PFS was 6.6 months (95%CI, 2.6–8,2 months) and median OS was 10.4 months (95%CI, 7.9–16.6 months).

An exploratory subset efficacy analysis was performed on 22 patients in this cohort who had non-refractory disease (i.e., excluding patients with CTFI ≤30 days after first-line therapy). All ten responses and seven SD occurred among these patients (ORR = 46% [95%CI, 24.4–67.8%]); DCR = 77% [95%CI, 54.6–92.2%]). Median PFS was 5.1 months (95% CI, 1.9–6.7 months) and median OS was 10.2 months (95% CI, 6.0–11.7 months) (Table 2).

### Safety

All treated patients were evaluable for safety. The most frequent treatment-related AEs or with unknown relationship were fatigue, nausea, decreased appetite, vomiting, and alopecia (Table 3). Most of these AEs were grade 1/2, with the most common grade ≥ 3 AEs being fatigue (25%), febrile neutropenia (14%) and nausea (7%). The only treatment-related AE to reach grade 4 was febrile neutropenia, in two patients. One episode of grade 5 treatment-related neutropenic infection occurred in one patient with ongoing diabetes, atrial fibrillation and chronic obstructive pulmonary disease, and disease involvement in the brain and lungs. No treatment-related cardiac adverse events associated with the LVEF occurred.

**Table 3:** Treatment-related adverse events (≥10% of patients or grade ≥ 3), and laboratory abnormalities regardless of relationship, in patients with relapsed SCLC treated with doxorubicin 40 mg/m^2^ and lurbinectedin 2.0 mg/m^2^ on Day 1 q3wk

	Doxorubicin 40 mg/m^2^ + lurbinectedin 2.0 mg/m^2^ (n = 28)							
	NCI-CTCAE grade							
	3		4		5		All	
	n	%	n	%	n	%	n	%
Treatment-related AEs								
Alopecia	.	.	.	.	.	.	7	25
Constipation	.	.	.	.	.	.	5	18
Decreased appetite	1	4	.	.	.	.	13	46
Dizziness	.	.	.	.	.	.	3	11
Dysesthesia	.	.	.	.	.	.	3	11
Dysgeusia	.	.	.	.	.	.	3	11
Fatigue	7	25	.	.	.	.	22	79
Febrile neutropenia	2	7	2	7	.	.	4	14
Hypertension	1	4	.	.	.	.	1	4
Mucositis	.	.	.	.	.	.	5	18
Nausea	2	7	.	.	.	.	19	68
Neutropenic infection	.	.	.	.	1	4	1	4
Vomiting	.	.	.	.	.	.	11	39
Hematological abnormalities								
Anemia	7	25	.	.	.	.	27	96
Leukopenia	14	50	9	32	.	.	26	93
Neutropenia	7	25	19	68	.	.	27	96
Thrombocytopenia	3	11	3	11	.	.	18	64
Biochemical abnormalities								
ALP increased	.	.	.	.	.	.	12	43
ALT increased	1	4	.	.	.	.	12	43
AST increased	.	.	1	4	.	.	9	32
Bilirubin increased	.	.	.	.	.	.	6	21
Creatinine increased	.	.	1	4	.	.	23	82

*AE*, adverse event; *ALP*, alkaline phosphatase; *ALT*, alanine aminotransferase; *AST*, aspartate aminotransferase; *NCI-CTCAE*, National Cancer Institute Common Terminology Criteria for Adverse Events; q3wk, every three weeks; *SCLC*, small cell lung cancer

Regardless of relationship, most laboratory abnormalities were grade 1/2. Grade ≥ 3 hematological abnormalities comprised anemia, neutropenia (grade 4 episodes lasting a median of 3 days [range, 1–6 days]), leukopenia, and thrombocytopenia (Table 3). The most common biochemical abnormalities were creatinine and transaminase increases. Grade ≥ 3 biochemical abnormalities consisted of transaminase increases in one patient with normal levels and no liver metastases at baseline, and grade 4 creatinine increase in one patient that was concomitant with severe renal failure unrelated to treatment.

Sixteen patients (57%) required granulocyte colony-stimulating factor support, seven (25%) were given red blood cell transfusions, and three (11%) received platelet transfusions. Eighteen dosing delays, seven lurbinectedin dose reductions and one doxorubicin dose reduction were the result of treatment-related AEs. Twenty-two patients died during the study, mostly (*n* = 21) due to disease progression and one due to treatment-related neutropenic infection.

The most common AEs related to single-agent lurbinectedin in the six patients who remained on treatment after doxorubicin discontinuation were fatigue (all patients), nausea (*n* = 4; 67%), dizziness and dyspnea (*n* = 3 each; 50%). Most of these AEs were grade 1/2. Grade 3/4 hematological abnormalities consisted of neutropenia, thrombocytopenia, leukopenia (50% of patients each), and anemia (33%). All biochemical abnormalities were grade 1.

## Discussion

A regimen of doxorubicin 40 mg/m^2^ and lurbinectedin 2.0 mg/m^2^ on Day 1 q3wk showed noteworthy antitumor activity in patients with relapsed SCLC included in an expansion cohort of a phase 1 study evaluating the doxorubicin/lurbinectedin combination in advanced solid tumors. ORR was 36%, with median DoR 5.2 months, median PFS 3.3 months and median OS 7.9 months. Most responses and longer median time-to-event parameters were found among patients with sensitive disease (ORR = 50%, DoR 5.5 months, PFS 5.7 months, OS 11.5 months). In contrast, patients with resistant disease only had one response (ORR = 10%, DoR 1.8 months) and shorter median survival times (PFS 1.3 months, OS 4.6 months). Of note, all responses occurred among patients with non-refractory disease (ORR = 46%, PFS 5.1 months, OS 10.2 months).

Treatment and survival of patients with SCLC has not changed substantially over the last two decades. Response rates to first-line therapy are high, but recurrence is frequent, especially in patients with extensive-stage disease [11, 12]. Topotecan is the only second-line therapy approved in the U.S. for patients with sensitive SCLC [12]. However, use of topotecan is challenging because of associated hematological toxicity and modest clinical benefit (ORR = 5–24%; median OS 6–8 months) [13–20]. Similar results have been reported for cyclophosphamide, doxorubicin and vincristine (CAV), a combination also used in second-line treatment of SCLC [13]. Alternative therapies such as monoclonal antibodies (nivolumab, atezolizumab) [21, 22] have also been evaluated for relapsed SCLC, but to date none have shown superiority over currently approved therapies.

The results obtained in this study with the doxorubicin/lurbinectedin combination compare favorably with those reported with second-line therapies for relapsed SCLC. Higher ORRs were achieved with the combination, both at the RD of doxorubicin 50 mg/m^2^ and lurbinectedin 4.0 mg FD defined during dose escalation (overall: 65%; sensitive disease: 89%; resistant disease: 38%) [7] and in the expansion cohort at the reduced dose of doxorubicin 40 mg/m^2^ and lurbinectedin 2.0 mg/m^2^ (overall: 36%; sensitive disease: 50%; resistant disease: 10%). Median survival times were also longer, especially in sensitive disease (PFS 5.8 months at the RD [7]; PFS 5.7 months and OS 11.5 months in the expansion cohort). In the expansion cohort, exclusion of patients with refractory disease (CTFI ≤30 days; a population usually not included in clinical trials due to having a poorer prognosis) increased the ORR to 46% and also resulted in long median survival times (PFS 5.1 months, OS 10.2 months). Of note, results from a cohort of 105 patients treated with single-agent lurbinectedin in a multicenter, multinational phase II Basket trial showed that lurbinectedin is active as second-line therapy in relapsed SCLC (ORR = 35%; median OS 9.3 months). Antitumor activity in this cohort was notable, both in sensitive (ORR = 45%) and resistant disease (ORR = 22%) [23, 24]. Of note, 20% of patients treated with single-agent lurbinectedin in this phase II Basket study had refractory disease (CTFI <30 days).

Treatment with the doxorubicin/lurbinectedin combination was generally well tolerated, and was primarily associated with manageable and predictable myelotoxicity. Compared with the RD defined during dose escalation, patients in the expanded cohort showed less grade 4 neutropenia (68% vs. 79%), grade 3 anemia (25% vs. 47%) and treatment-related febrile neutropenia (14% vs. 26%) [7]. Biochemical abnormalities were mostly mild or moderate and asymptomatic, and occurred at similar frequencies in the two cohorts. Some treatment-related non-hematological adverse events (mucositis, 18% vs. 53%; alopecia, 25% vs. 42%) were less common in the expansion cohort than at the RD, while others occurred at similar frequencies (fatigue, 79% in both cohorts; nausea/vomiting, 39–68% vs. 58%; decreased appetite, 46% vs. 53%) [7]. No patients in either cohort discontinued treatment with the combination due to treatment-related events. Of note, the absence of treatment-related cardiac events associated with the LVEF either during dose escalation or in the expansion cohort suggested that lurbinectedin does not increase ventricular dysfunction over doxorubicin. The safety profile of single-agent lurbinectedin in the expansion cohort is in agreement with that reported elsewhere [5, 23].

In summary, remarkable antitumor activity has been found for a combination of doxorubicin 40 mg/m^2^ and lurbinectedin 2.0 mg/m^2^ on Day 1 q3wk in an expanded cohort of patients with relapsed SCLC, particularly in sensitive disease, and higher than that reported for currently approved second-line therapies. Compared to this dose, the initial RD defined in this study (doxorubicin 50 mg/m^2^ and lurbinectedin 4.0 mg FD) resulted in a higher ORR but also higher toxicity, with more frequent hematological abnormalities and treatment-related mucositis and alopecia. Of note, single-agent lurbinectedin has recently been approved by the U.S. Food and Drug Administration for the treatment of adult patients with metastatic SCLC with disease progression on or after platinum-based chemotherapy. An ongoing open-label, randomized phase III trial is evaluating the doxorubicin/lurbinectedin combination vs. standard-of-care chemotherapy (CAV or topotecan) in patients with SCLC and CTFI ≥30 days that has progressed after one line of platinum-based chemotherapy [25].

## Data Availability

Individual participant data are not publicly available since this requirement was not anticipated in the study protocol considering that this trial started patient enrolment in 2011. Clinical trial summary results were placed in the European Clinical Trials Database (EudraCT; https://eudract.ema.europa.eu).
